# Controlled Formation of Carbon Nanotubes and Nanotube Junctions from Bilayer Graphene

**DOI:** 10.1002/smll.202513639

**Published:** 2026-03-09

**Authors:** Michael Schlegel, Mitisha Jain, Arkady V. Krasheninnikov, Jannik C. Meyer

**Affiliations:** ^1^ University of Tübingen Institute of Applied Physics Tübingen Germany; ^2^ NMI Natural and Medical Sciences Institute at the University of Tübingen Reutlingen Germany; ^3^ Institute of Ion Beam Physics and Materials Research Helmholtz‐Zentrum Dresden‐Rossendorf Dresden Germany

**Keywords:** carbon nanotubes, electron microscopy, graphene, nanofabrication

## Abstract

$Sp^{2}$ bonded carbon structures exhibit a rich variety of morphologies depending on how the graphene as basic unit is laterally constrained and topologically connected to itself. Here, we demonstrate that the connection that forms between two adjacent layers of graphene after cutting by focused electron beam can be exploited in a controlled fashion to induce a spontaneous reconstruction from the flat to a tubular or even more complex geometry. In particular, we demonstrate the cutting of twisted bilayer graphene to create chiral carbon nanotubes (CNTs), using a scanning transmission electron microscope operated at 200 kV and with line doses of $10^{9}$ electrons per nanometer. By choosing the cutting angle halfway between the two graphene orientations, a seamless tube could be formed in principle, and relatively straight tube sections are obtained in practice. Bilayer graphene ribbons with a width of less than approximately 4 nm spontaneously convert to CNTs, while no nanotubes are formed from wider ribbons. Moreover, CNT arrays and CNT junctions are prepared in a controlled way. The transformations from a nanoribbon to a nanotube are also reproduced via analytical potential molecular dynamics simulations. Devices made from such junctions could be useful for nanoelectronics, quantum transport, interconnects or nanofluidics.

## Graphical abstract

Twisted bilayer graphene is cut using a scanning transmission electron microscope to create individual chiral carbon nanotubes and interconnected nanotube junctions. The sculpting is facilitated by in situ heating and automated beam control. Bilayer graphene ribbons with a width of less than approximately 4 nm spontaneously convert to CNTs, while no nanotubes are formed from wider ribbons, in agreement with molecular dynamics simulations.

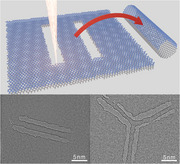

## 1 Introduction

**Figure 1:**
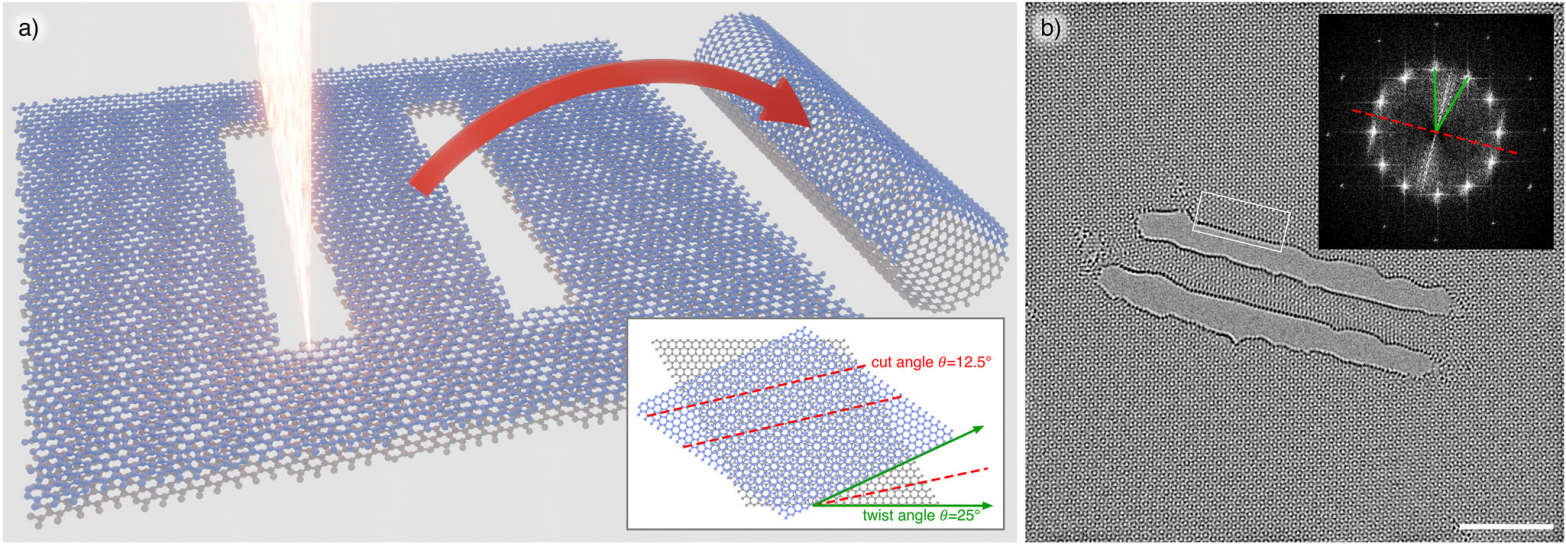
a) Schematic representation of the CNT fabrication process, as two parallel lines are cut into twisted BLG to produce a nanoribbon whose edges then coalesce to form a chiral nanotube. The inset illustrates the relationship between the twist and cut angle required for the formation of a chiral CNT. b) High resolution transmission electron microscopy (HRTEM) image of a CNT fabricated in this way. The white rectangle indicates the position of the close‐up shown in Figure 4f. The inset shows the Fourier transform. The green lines indicate the relative twist angle between the graphene sheets. The red dashed line shows the cut angle and tube orientation in real space. Scale bar is 5 nm.

The ongoing miniaturization across a wide range of technological devices is inherently connected to the continuous improvements in nanofabrication methods [1]. In this context, low‐dimensional materials and their modifications are promising ingredients for nanoscale circuitry [2], nanofluidics [3], sensing [4], or nanoscale optics [5]. $Sp^{2}$ bonded carbon forms a rich variety of allotropes of different dimensionalities, such as fullerenes (0D), carbon nanotubes (1D), graphene (2D), or graphite (3D). The transformation among carbon allotropes of different dimensionalities [6] has already been studied to some extent: for example, fullerenes (0D) can coalesce into nanotubes (1D) [7], nanotubes can be unzipped to form monolayer graphene ribbons (2D) [8, 9], and inversely, graphene nanoribbons can be rolled up into nanotubes [10]. Furthermore, the collapse of large‐diameter carbon nanotubes (CNTs) into bilayer graphene ribbons has been described in several works [11, 12, 13, 14, 15, 16, 17, 18]. Whether the structure remains circular in the cross‐section or becomes flat depends on the interplay of the van der Waals (vdW) interaction of the opposing walls and the elastic bending energy of the tube. The formation of CNTs from graphene was previously demonstrated by cutting multi‐layer graphene and subsequent thinning by broad electron irradiation at an elevated temperature [19], and similarly for hexagonal boron nitride [20], as well as from AA‐stacked bilayer graphene (BLG) at room temperature [21]. These processes appear to be challenging to control in terms of nanotube length and diameter, and with the latter suffering from contamination and producing rather short and irregular tube segments. Here, we show a more controlled pathway to form chiral CNTs from twisted BLG, as well as CNT arrays and junctions. Contrary to single‐crystalline BLG, where a cut along the armchair or zigzag direction produces two identical nanoribbons, a similar cut in twisted BLG yields two ribbons with differing orientations. These ribbons cannot seamlessly merge at their edges since they are geometrically incompatible. However, when the twisted BLG is cut along a high symmetry axis, e.g., along a symmetry axis of the moiré pattern, the two nanoribbons exhibit mirror symmetry with respect to each other, and their edges become structurally compatible. The dangling bonds of each ribbon can then recombine to re‐form hexagons, enabling the two ribbons to merge seamlessly into a chiral CNT. Figure 1a provides a schematic illustration of the concept. These conditions can be realized experimentally by cutting the BLG at exactly half of its twist angle. Moreover, by the controlled patterning of graphene, it is possible to define multiple CNTs in desired locations and even connected nanotubes. As we demonstrate, Y‐junctions of CNTs can be formed, where the graphene layers become separated throughout the junction, thus forming a connection with an open interior space. Hence, besides electronic applications, such Y‐junctions may be useful for nanofluidics on the smallest scale. Indeed, the unfolding of sculpted bilayers beyond the simple tubular geometry opens the door for a rich variety of complex molecular structures by atomic level kirigami, such as those theoretically studied in Refs [22, 23].

Various methods have been employed to structure 2D materials. These include, for example, the use of a focused ion beam (FIB) [24], electron beam lithography [25], or scanning probe lithography [26]. In this work, we employ the focused electron beam of a scanning transmission electron microscope (STEM), which represents another promising candidate for structuring 2D materials [27, 28, 29, 30]. When operated at 200 kV, the electrons possess enough energy to ballistically displace atoms from the lattice [31, 32]. Compared to other methods, it is slow and lacks scalability. However, it provides an unprecedented structuring resolution and is thus the unchallenged method of choice for structuring at nanometer length scales. While the aforementioned knock‐on damage pathway resembles the main pathway in which the structure of the material is modified at high accelerating voltages, there are a number of additional mechanisms happening at the same time. Inelastic scattering events with atoms of adsorbates on the sample or with residual gases within the microscope can lead to their ionization. The thereby generated reactive species can then lead to chemical etching of the 2D material [33, 34]. While this might resemble a way to structure the material on its own, it is less controllable and thus an undesirable process here. Moreover, surface adsorbates and general sample contamination constitute a problem for electron beam‐based direct structuring. In particular, if the typical hydrocarbon contamination is exposed to the electron beam, the hydrocarbons are cracked, so that the hydrogen is dissipated and the carbon residuals form amorphous carbon. This then becomes permanently attached to the sample surface and cannot easily be removed. To prevent contamination and ensure an atomically clean surface, we employed in situ heating chips as a substrate. Indeed, it turned out to be imperative that the sample is at high temperatures whenever it is exposed to an electron beam, as otherwise amorphous carbon would immediately attach, especially at open bonds or defects.

## 2 Methods

### 2.1 Sample Preparation

As a substrate, an in situ heating chip was utilized. The chip, as well as a special sample holder, were purchased from HennyZ. The membrane of the heater chip was perforated using the FIB. Two layers of chemical vapor deposition grown graphene were deposited, one after the other, using the Graphene Easy Transfer system supplied by Graphenea. This process covers the holes of the heater chip with freestanding, randomly twisted BLG. Due to the finite (μm‐scale) grain size of the graphene, many different twist angles can now be found in different locations on the sample.

### 2.2 STEM Structuring and HRTEM Imaging

All transmission electron microscopy (TEM) and STEM work was carried out using an image‐side aberration‐corrected Jeol ARM200F. The sample was kept at an elevated temperature (+500°C) during all TEM and STEM work. As preparation for cutting the BLG at the correct angle, the twist angles were determined beforehand. For this purpose, the sample was investigated at 80 kV in high‐resolution TEM (HRTEM) mode to locate large clean areas and measure their twist angle in Fourier transforms of the image. To cut the twisted BLG into nanoribbons, the microscope was operated in STEM mode at 200 kV. The probe was estimated to have an effective diameter of 1 nm, based on the edge sharpness of contamination metal particles on the sample imaged just before or after cutting. Since the regions of interest were located at 80 kV, tuning of the 200 kV beam could be performed away from this area, thus minimizing the exposure to the high‐energy electrons. In preliminary tests, a suitable dose that would allow for effective milling of the bilayer graphene was empirically determined. For the cutting, the beam was positioned at predefined coordinates on the sample, mediated by an automated script. Line cuts were thereby defined as points that are spaced 0.25 nm apart. The line dose is estimated to be $9.7\times 10^{8}\;e^{-}/\text{nm}$, based on the current measured on the fluorescent screen of the TEM. In this fashion, nanoribbons were produced by cutting pairs of parallel lines, as also illustrated in Figure 1a. To obtain CNTs of varying diameter and length, the spacing between the cuts and their respective length was altered. The individual cuts were additionally broken up into smaller 5 nm sections. The cuts were carried out alternately on each side of the ribbon to potentially reduce the risk of the structure bending and attaching to the BLG edges. For the Y‐junctions, a sequential clockwise approach was used. This approach resulted in three bilayer ribbons spaced 120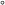 apart. Based on the rotational symmetry of BLG, this theoretically yields three identical chiral CNTs connected in the middle. A more detailed discussion about the cutting sequences of the different structures is provided in Figure S3 (Supporting Information). After completion of the patterning process, the script automatically closes the beam valve to minimize further beam‐induced damage.The microscope was then again operated at 80 kV in HRTEM mode for imaging. Aberration‐corrected HRTEM images were obtained with a spherical aberration of ca. +5 μm and a defocus of ca. –8 nm, so that dark contrast corresponds to atomic structure.

### 2.3 Atomistic Simulations

All analytical potential molecular dynamics (MD) simulations were performed using the LAMMPS code [35]. To describe the C–C interactions in the carbon structures, the adaptive intermolecular reactive empirical bond order (AIREBO) potential  [36, 37, 38] was utilized. In the first step, two graphene layers were placed on top of each other at a distance of about 3.3 Å with a certain twist angle (θ=11.6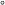, 27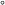) between them. Following that, the optimized twisted BLG was cut to form ribbons of different widths and edges. Both top and bottom layers were either cut along the axis of half of the twist angles, as attempted in the experiment, or along a direction that does not coincide with a symmetry axis in order to assess the effect of a miscut. In the final step, the systems were thermalized at high temperature (T=2000 K) in the NVT (canonical) ensemble. The thermalization simulation time ranged between 50 and 100 ps, with timesteps of 0.1 fs. The atoms at the two ends of the nanoribbons were fixed during the simulation run. Based on the atomic structures obtained in these calculations, HRTEM image simulations were conducted using the abTEM Python package.

## 3 Results

**Figure 2:**
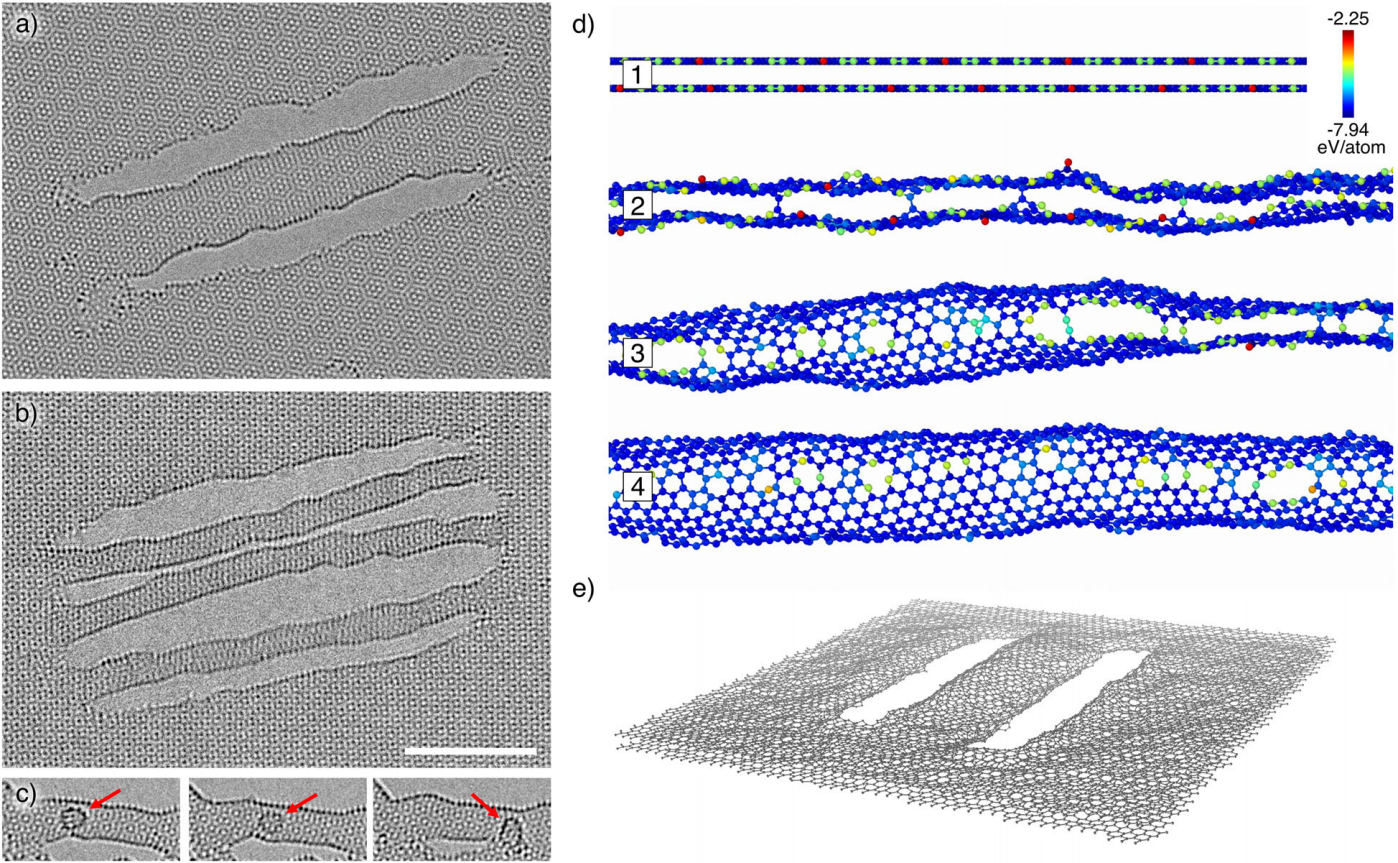
a) CNT made from BLG with a chiral angle of approximately 22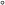. b) Array of three CNTs next to each other. Scale bar is 5 nm and applies also to (a–c). c) Time series of a fullerene‐like structure (red arrow) moving through the inside of a tube. d) Time series of a MD simulation of a BLG ribbon with open transforming into a nanotube (only the front half of the tube is shown for clarity). e) 3D perspective view of a nanotube structure and surrounding graphene as obtained via MD simulations.

**Figure 3:**
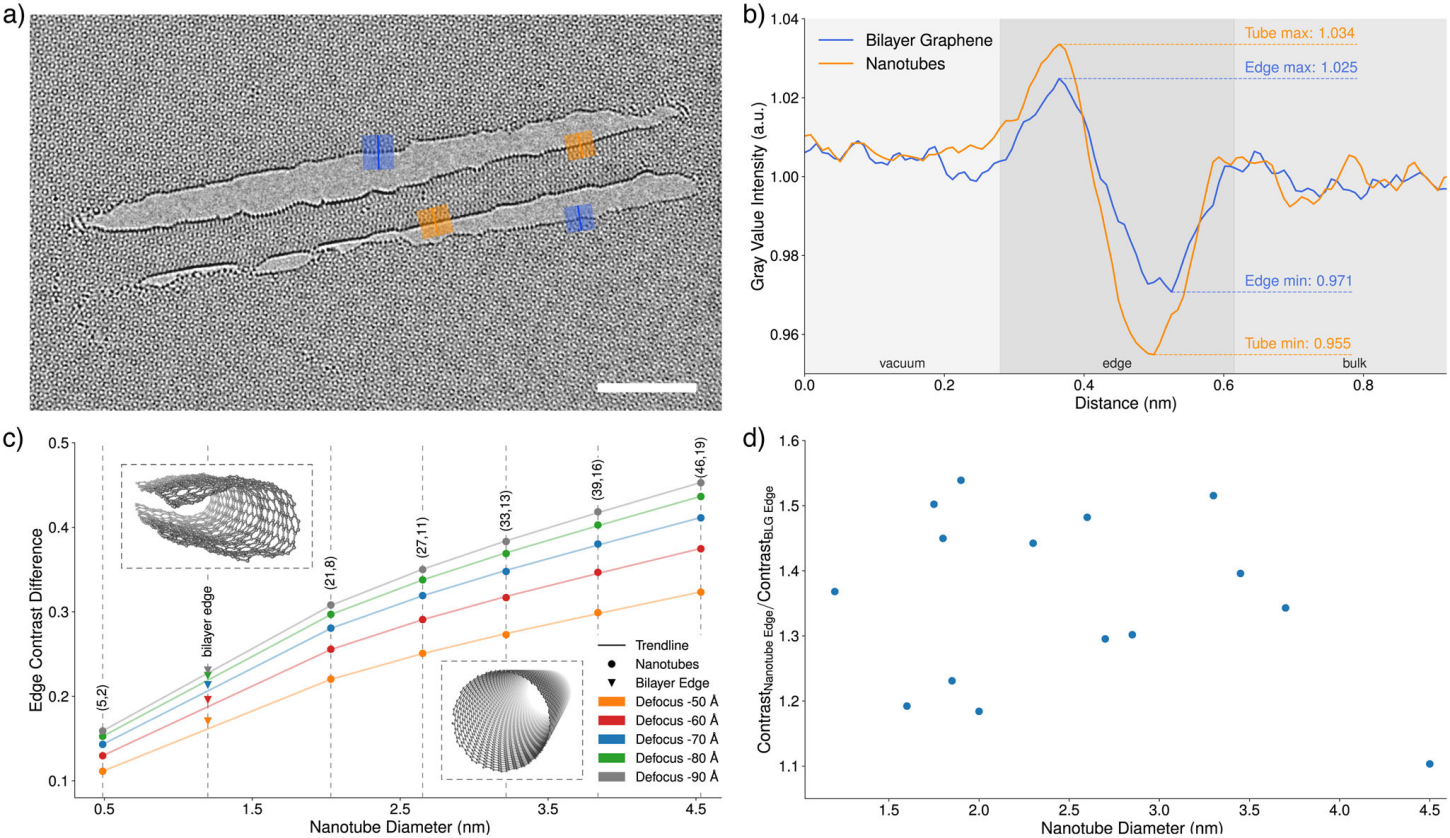
a) Edge contrast HRTEM image of a fabricated CNT and the positions of the line profiles used in (b). Scale bar is 5 nm. b) Plot of gray value intensity changes across structure edges. The bilayer graphene curve corresponds to the average of the blue line profiles, while the nanotube curve shows the average of the two orange line profiles of the nanotube in (a). c) Contrast differences at the edges of simulated chiral CNTs for different defoci. The contrast difference was calculated by subtracting the minimum from the maximum gray value intensity of the edge line profile. Additionally, the contrast change of a simulated bilayer edge is shown for comparison. d) Relative contrast difference between CNT and BLG edges of the fabricated structures.

**Figure 4:**
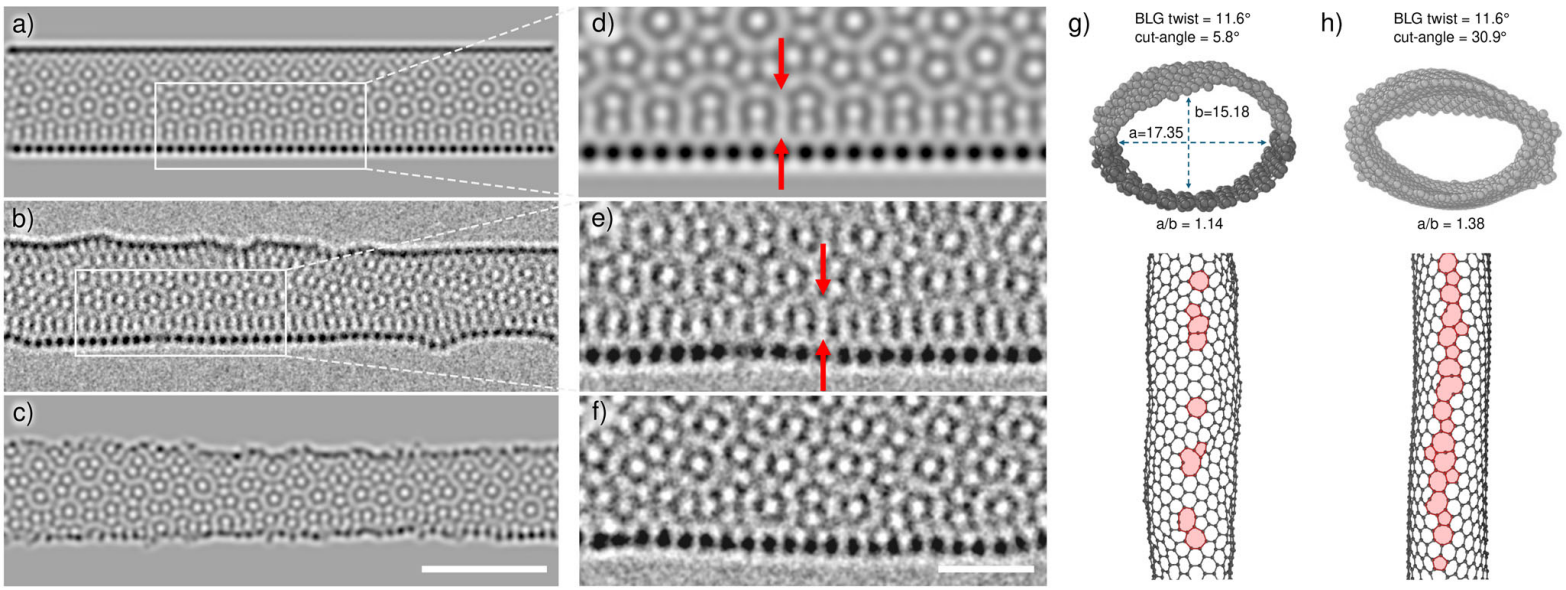
a) Simulated TEM image of chiral CNT (21,8). b) Real HRTEM image of a fabricated tube. c) Simulated TEM image of a CNT formed by MD simulation. d)+e) Close‐up view of (a) and (b) showing a characteristic appearance of a rounded edge indicated by the red arrows. f) Close‐up of a BLG edge taken from Figure 1b not showing the same characteristics. g) MD‐simulated nanotube, formed by cutting at the correct angle. h) MD‐simulated nanotube, formed by cutting at the incorrect angle. The incorrectly cut CNT is less round (higher a/b ratio) and shows a higher defect density at its tube wall in the side view below. Non‐hexagonal rings in the lattice are colored in red. Scale bar is 2.5 nm (a–c) and 1 nm (d–f).

Examples of the fabricated CNTs are presented in Figures 1, 2, and 3a. Overview images of more CNTs of different lengths and diameters are provided in Figure S1 (Supporting Information). Besides the CNTs, the images also show that the surrounding BLG is virtually free from contamination, which is a necessity when trying to structure at these length scales. As previously mentioned in the introduction, it is of great importance to cut the BLG at half of its twist angle in order to obtain two structurally compatible nanoribbons. Verification of the successful execution of this process within the experiment is shown in the inset of Figure 1b. The two green lines indicate the twist angle between the BLG, and the red line shows the cutting direction in real space. Figure 2c shows a fullerene‐like structure that presumably formed from residual carbon trapped inside a fabricated nanotube. The fullerene can be seen moving inside the nanotube along the tube axis within Movie S1 (Supporting Information). Figure 2d showcases a series of snapshots from the MD simulation, illustrating the typical tube formation from a bilayer nanoribbon. The complete simulation is available in Movie S2 (Supporting Information). Figure 2e presents an illustration of the final structure. The open edges of the bilayer have closed up and the ribbon has transformed into a nanotube, although somewhat flattened. In order to verify the tube characteristic of the fabricated structures, we analyzed the contrast intensity of the respective structure edges. Under our imaging conditions, the contrast of the curved carbon sheet increases proportionally with the amount of material traversed by the electron beam. Thus, because of its lower curvature, the contrast will be stronger for the edge of a CNT compared to the edge of a flat nanoribbon. To quantify this, line profiles were taken of the structures of interest, measuring the change in gray value intensity. Figure 3b shows such line profiles, which were taken from Figure 3a. The measurement direction is from vacuum to edge. Thus, the line first shows a peak in intensity resulting from the Fresnel fringes of the structure edge, followed by a minimum. We define the contrast difference as the maximum gray value intensity minus the minimum, normalized to the mean intensity in the image. The plot shows that this difference is higher for the CNT edge compared to the BLG edge, indicating an increased edge height for the CNT. In Figure 3c, the contrast differences at the edge of chiral CNTs within simulated TEM images are shown. As expected, a larger tube diameter, which is accompanied by a higher number of atoms within the viewing direction, results in a stronger contrast difference at the respective tube edge. The plot also shows that the contrast depends strongly on the applied defocus. Furthermore, contrast measurements of a simulated BLG with merged edges are provided. The height of the edge is roughly 1.2 nm and fits well with the trend line, calculated from the CNT values. Figure 3d shows contrast ratios obtained from the experimental data. Each data point represents measurements taken from one image. Within each image, two to four line profiles were measured as indicated in Figure 3a, followed by the calculation of the contrast difference as exemplified in Figure 3b. The values for the two line profiles of the CNT edge were averaged, as well as the two values of the BLG edge. Finally, the ratio of the CNT contrast and the BLG edge contrast is calculated and given as a data point. This results in a relative measure that shows how much stronger the contrast change is at the CNT edge compared to the BLG edge within the same image (and hence imaged at the same defocus). Despite the considerable spread of the data, which we attribute to deviations from the ideal tube structure as well as to variations in defocus and other imaging parameters, all the points except for the largest diameter (4.5 nm) are consistently well above unity. This shows that all measured CNTs exhibit a stronger change at the edge than at the respective bilayer edge. One would also expect the ratio to increase for larger tubes; however, this trend can not be clearly verified due to the spread of the data points. Finally, the data point for the 4.5 nm ribbon is close to 1 and below all other data points, indicating that this wide ribbon did not unfold into a nanotube. From a geometrical perspective, a 4.5 nm wide ribbon would be expected to unfold into a 3.5 nm nanotube, while the largest tube that we observed had a diameter of 3.7 nm. Hence, we can identify the limit for the transition between the collapsed and open tube to be in the range of 3.5–3.7 nm. Figure 4a–c compares HRTEM simulations of an ideal chiral CNT (a) with an HRTEM image of the fabricated nanotube (b) (a section of the nanotube from Figure 1b and a HRTEM simulation of a tube from the MD simulation (c). The structures in images (a) and (b) were tilted by 10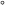 prior to the simulation to better match the appearance of the experimental tube, where the lattice spacing is visible in the bottom tube wall due to the matching direction of the projection. While the perfect CNT and the experimental one both have straight edges, the MD‐simulated tube shows a more rough outline. Figure 4d–f provides a close‐up comparison between the edges of the simulated nanotube, the experimentally obtained one, and an edge of the bilayer graphene next to the same nanotube. The red arrows in (d) and (e) indicate a distinctive appearance caused by the low curvature of the nanotube edges, a feature not observed in (f) for the BLG edge: In the CNT images, the moire pattern near the center is different from that close to the edges of the tube; while for the BLG graphene, the moire pattern appears to be the same throughout, simply truncated at the edge. To quantitatively compare the roundness of the simulated tubes, we introduce the a/b ratio, which is the height of the tube a divided by its width b. Figure 4g,h presents cross‐sections of MD‐simulated CNTs, produced by both correct and incorrect cut angles alongside their respective a/b ratios of 1.14 and 1.38. Furthermore, a side view of the CNTs is provided, illustrating the defects formed during tube formation. It shows that the incorrect cutting angle has led to a higher defect density along the connecting seam, which is consistent with the higher a/b ratio and the rough outline in the TEM simulation.

**Figure 5:**
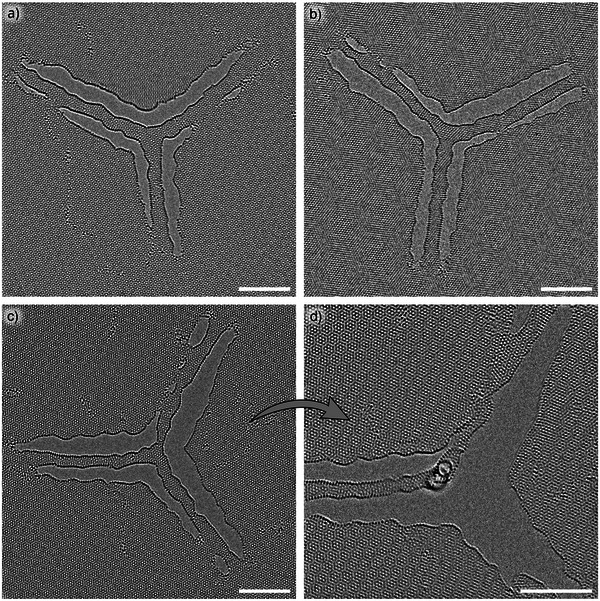
a–c) Examples of the fabricated CNT Y‐junctions. d) Y‐Junction also shown in (c), which snapped off at the lower branch after beam exposure. The capped branch bent upward, allowing a view along the round cross‐section of the CNT. Scale bars are 5 nm.

To demonstrate a structure that is more complex than a tubular shape, we made Y‐junctions between three CNTs. Figure 5a,b shows examples of the produced nanotubes and their junctions. For the most part, the junctions are freestanding. However, some tube sections have also attached to the BLG. Images (c) and (d) were taken just shortly after each other. During TEM observation, one of the tubes next to the junction snapped and bent upwards. As a result, a view through the circular cross‐section of the nanotube was visible.

## 4 Discussion

The HRTEM images from the various CNTs produced in the experiment demonstrate that the diameter and length of the fabricated CNTs can be controlled by adjusting the spacing and length of the parallel cuts. The twisted BLG is cut at an angle of half the twist angle to promote a seamless edge reconstruction. This ensures that the top and bottom layers have the same density of dangling bonds per unit length, and thus the two layers of the ribbon can connect to each other and form a tubular structure with a potentially very low density of defects at the seam. High‐temperature annealing may further reduce the number of defects at the seams. However, it must be noted that for a perfect chiral nanotube, only discrete values of chiral angle and diameter corresponding to a pair of integer indices (n,m) of a CNT would be permitted. Hence, by using randomly twisted BLG and an arbitrarily chosen ribbon width, the produced CNTs will exhibit a chiral angle and diameter which will be close to those of a valid pair of indices (n,m), while some small mismatch inevitably remains. In the experiment, the length was limited by the dynamic movement of the tubes, which eventually attached to the surrounding structures. However, this should be controllable by removing more material to the sides of the CNTs. It was argued previously that tubes with increasing diameter eventually collapse into flat bilayer nanoribbons [11, 12, 13, 14, 15, 16, 17]. In our work, we make use of the opposite mechanism ‐ sufficiently narrow bilayer nanoribbons “un‐collapse” into nanotubes. Both processes depend on the balance between the bending energy of the graphene sheet and the vdW interaction between the layers, and hence, a similar threshold diameter can be expected. Nevertheless, it is interesting to note that, in our case, the bonds that form between the two graphene layers at their open edges [39, 40] apparently form in such a way that this transition is initiated, implying that the bond is highly distorted upon formation and then relaxes toward the tubular shape. The reported threshold diameters for which the collapsed configuration of a CNT is favored range from 1.9 to 7.0 nm, depending on the methodology and parameters employed by the respective works [11, 12, 13, 14, 15, 16, 17, 18, 41]. This is in line with our own simulations, which indicate that tubes of a diameter of above 4 nm will remain as flat ribbons and do not unfold into nanotubes, although the edges do form a curved connection between the layers. In our experiments, no CNT could be produced with a diameter exceeding 4.5 nm. Experiments and simulations for these larger ribbons are given in the Supporting Information. An energetic analysis following the arguments of Chopra et al. [17], shown in Figure 6 (Supporting Information), illustrates the critical radius for collapse based on the energy per unit length. In addition, it should be noted, that the collapse of a nanotube into a flat ribbon and the reverse process (“un‐collapsing”) will likely involve a kinetic barrier arising from attractive vdW interactions between the graphene layers, such that metastable configurations could persist even if they are not energetically favored [41]. To overcome this kinetic barrier, temperature may play a crucial role. MD simulations have shown that the collapse diameter of CNTs increases significantly at elevated temperatures [42]. Since all our observations were made at temperatures above 500 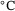, both thermal effects and beam irradiation are likely to have facilitated the unfolding of the bilayer ribbons. Furthermore, it was suggested that CNTs do not collapse simultaneously along the whole CNT length but rather at local points, which then propagate through the tube [43]. In our case, the nanotube end points are forced to be flat by the adjacent bilayer graphene, and the transition from flat to tubular occurs over a short length (ca. 1–2 nm, estimated by the change in contrast from the BLG edge to the stronger contrast of the CNT wall). Thus, the tubes that we observe must be stable against collapse even in the presence of the forced flat configuration at the end. Finally, the structure of the as‐formed nanotubes along the seam formed by connecting the two layers deserves a careful discussion. In the MD simulation, new bonds form between the two layers and the nanotube unfolds, but a significant amount of defects remains (Figures 2d and 4g). Figure 4 shows a comparison between a simulated image for an ideal CNT, the experimental image, and a simulated image for the nanotube generated from the bilayer ribbon by MD simulations. The apparent roughness of the CNT walls in the experiment is somewhere in‐between the two simulated cases. This suggests that the walls in the experimentally formed tubes are less defective than those formed by MD simulations, while still being more irregular than the perfect CNT. This is further confirmed by the cross‐section view obtained in Figure 5d where a circular cross‐section can be seen, while the CNTs formed by MD simulations are always somewhat elliptical. The overall higher defect density within the simulated structures might be explained by the limited simulation time. While the real CNTs were annealed at elevated temperatures during the entire observation time (several hours), the simulations only covered 50–100 ps, although at 2000 K. Figure 4g,h shows that there can be a variety of defects within the tube walls, some of which may be removed more easily than others: If the edges were formed by a cut at half of the twist angle, there will be the same number of open bonds per length in both layers, resulting in point‐defect like structures along the connection (Figure 4g) which might be annealed out (although some strain will remain if the angle and ribbon width do not correspond to one of the discrete pairs of angle and circumference of a valid nanotube). However, in instances where the edges are incompatible to begin with (Figure 4h), the connections between the layers will be similar to a grain boundary in graphene (a series of unpaired dislocation cores), which leads to local distortions [44] and can not be easily removed by annealing. An example of defect annealing within a CNT wall is shown in Figure S5 (Supporting Information). Defects may also be introduced by the high energy electron irradiation, in particular during the 200 kV structuring. While precautions were taken to minimize exposure to 200 kV electrons, as described in the methods section, some unintended exposure is unavoidable. This includes scanning mode imaging required to move to the region of interest, as well as beam tail induced damage to the surrounding structures during patterning. Inevitably, this results in some defect formation within the graphene and may make seamless coalescence of the nanoribbon edges more challenging. However, it appears that the elevated temperatures in the experiment can significantly heal out those defects, as it was already shown for graphene [19, 45], so that the observed graphene and nanotube lattices away from the edges of the cuts remain largely free from point defects. Figures 2c and 5 show further capabilities of our approach. The nanostructuring of BLG allows the precise placement of nanotubes at desired locations, enabling their further interconnection. Figure 2b demonstrates the formation of an array of CNTs. Based on the cutting pattern, these three CNTs should be identical. However, one can see that the upper two tubes stick to each other along part of the length, forming a CNT bundle with the two walls separated by a distance that suggests vdW adhesion, rather than covalent bonds. We also observe that during TEM imaging CNTs oscillate and deform. It appears likely that the bilayer graphene and the nanotubes already start to deform during the cutting process, e.g., due to edge closure, vdW adhesion, or beam‐induced defects. For this reason, different cutting sequences were explored, as described in the Supporting Information. Multiple CNTs can not only be lined up next to each other, but also can be connected to each other (Figure 5). In this way, nanotube junctions [46], which are directly connected via a graphene‐based, $sp^{2}$‐bonded carbon structure, are formed. Since the edge of the tube does not change its appearance across the junction, we conclude that the two graphene sheets are also separated through the junction, leaving an open space within the junction that connects the interior hollow space of the CNTs. Again, these structures are more sensitive to the electron beam than pristine graphene, and the connection between the three CNTs in the middle hangs freely between three tubes. Thus, the structure moves easily during observation, and the side walls of the tubes partially reattach to the surrounding graphene edge. A partly broken structure provided a cross‐sectional view of the junction (Figure 5; Movie S3, Supporting Information): It confirms that the nanotube has a nearly circular cross‐section, and is also in agreement with an open space in the junction volume. The difficulty of controllable placement, selection of chirality, and connection of CNTs is arguably the main obstacle for creating nanoscale electronic circuits from CNTs [47]. Carving CNTs out of twisted BLG may contribute to overcoming these obstacles, as we have demonstrated in this work. Less defective CNTs, possibly by improved annealing [48], would be desirable. Nanotubes with a connected interior space at the junction might also offer intriguing options for mass transport within the (branched) channel, e.g., for nanofluidics, or to facilitate reactions between two ingredients in a confined space [49, 50]. On a more general note, we have demonstrated that the tendency of graphene edges to connect and unfold into curved structures can be exploited for targeted nanofabrication. This directly opens new avenues for the sculpting of molecular‐scale structures [22, 23], and may also be a useful ingredient for a (slightly larger scale) nano‐kirigami/origami with 2D materials [51, 52].

## 5 Conclusion

We have demonstrated that chiral CNTs, CNT arrays, and CNT junctions can be fabricated from twisted BLG by electron beam sculpting in STEM, facilitated by in situ heating and automated beam control. The technique can consistently fabricate chiral CNTs of different sizes exhibiting a genuine tubular characteristic, surpassing the roundness of simulated counterparts. The ribbon must be cut at an angle of half the BLG twist angle; incorrect cut angles induce numerous defects in the seam areas, reducing the roundness of the structure. Both simulation and experiment confirmed that wide diameter ribbons of 4.5 nm or more remain as flat nanoribbons. The ability to produce interconnected structures with high precision may be useful for next generation nanoelectronic devices, sensors or nanofluidic systems. The conversion from flat to curved graphene, driven by the tendency of edges to close dangling bonds, opens the door to nanostructure definition by nanoscale‐kirigami techniques and could easily be extended to more complex structures by the use of modified writing patterns or the extension to other layered materials.

## Conflicts of Interest

The authors declare no conflict of interest.

## Supporting information


**Supporting File 1**: smll73030‐sup‐0001‐SuppMat.pdf.

**Supporting File 2**: smll73030‐sup‐0002‐Movie1.mp4.

**Supporting File 3**: smll73030‐sup‐0003‐Movie2.mp4.

**Supporting File 4**: smll73030‐sup‐0004‐Movie3.mp4.

## Data Availability

The data that support the findings of this study are available from the corresponding author upon reasonable request.
