# Supplementary material for: Controlled Formation of Carbon Nanotubes and Nanotube Junctions from Bilayer Graphene
Source: Small. 2026 Mar 9;22(26):e13639. doi: 10.1002/smll.202513639 (PMC13155060; doi:10.1002/smll.202513639)
Supplement: Supplementary file 1 — Supporting File 1: smll73030‐sup‐0001‐SuppMat.pdf. [file SMLL-22-e13639-s004.pdf]

Supporting Information

Controlled formation of carbon nanotubes and nanotube junctions from bilayer graphene

Michael Schlegel[1,2], Mitisha Jain[3], Arkady V. Krasheninnikov[3], and Jannik C. Meyer[1,2]

[1]University of Tübingen, Institute of Applied Physics, 72076 Tübingen, Germany

[2]NMI Natural and Medical Sciences Institute at the University of Tübingen, 72770 Reutlingen, Germany

[3]Institute of Ion Beam Physics and Materials Research, Helmholtz-Zentrum Dresden-Rossendorf, 01328 Dresden, Germany

1. Additional Experimental Information

Fig.1 shows four overview images of structures fabricated with different parameters. Subfigure (a) demonstrates the variety of nanotubes with different widths and lengths, which can be produced by changing the cut spacings and lengths. Furthermore, it is evident from the image that longer tubes are less stable and attach more frequently to the surrounding bilayer edges than their shorter counterparts. Within (b), the limiting cases are shown. If the cuts are spaced too closely, no bilayer nanoribbon will form because the electron-beam paths overlap due to the beam dimensions. When the cuts are spaced more than 5 nm apart, carbon nanotube (CNT) formation does not occur. In fact, we do not even observe well defined ribbons in that case, which is further discussed below. The images also show how clean the graphene has become as a result of heating, with only a few isolated contamination clusters remaining. Reproducibility was especially high for tubes with lengths of 10–20 nm and widths of 1.3–2.5 nm as represented by the CNTs within row 2-3 and column 2-3 of images (b) and (c)

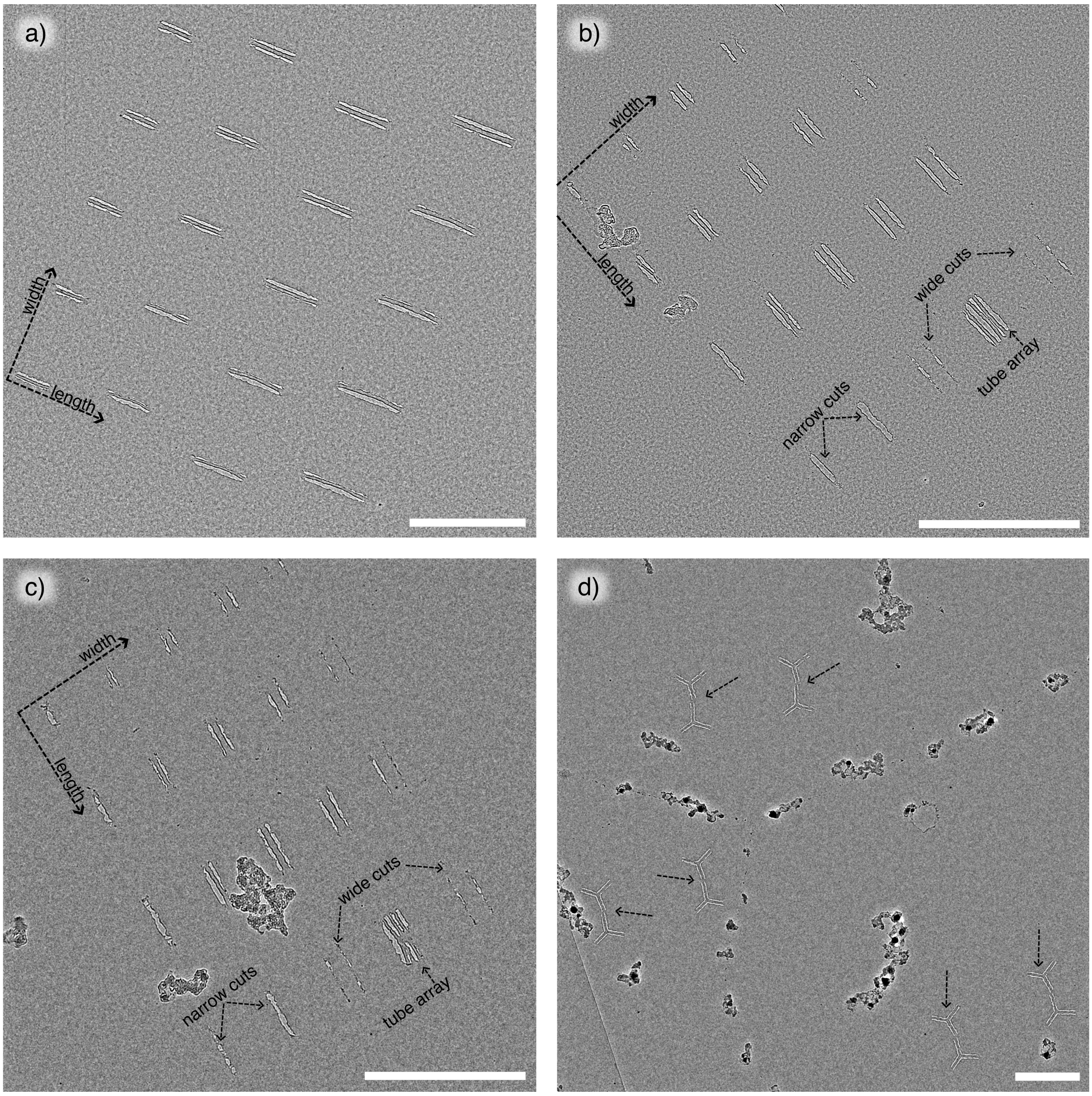

FIGURE 1 a) Overview TEM image of the fabricated CNTs with increasing tube width from left to right and increasing length from top to bottom of the pattern. The nanotubes were cut using two straight parallel cuts. b) Overview image of CNTs with more parameter variation. The first three rows are with increasing width and length. The bottom row includes a successful fabrication of a nanotube array as well as 2 extremely narrow and two wide cuts, which did not lead to CNT formation. c) Same pattern as in (b) executed elsewhere on the sample. In (b) and (c), the line cuts were split up into alternating 5 nm sections. d) Overview image of several Y-junctions, written with the cutting sequence shown in Fig. 3. While most junctions were fabricated successfully, the connection between adjacent junctions displays an offset which is attributed to sample drift. Scale bars are 50 nm.

To optimize the quality of the fabricated structures, different cutting approaches were chosen based on the requirements, as illustrated in Fig. 3. In the preliminary tests and in general for shorter tubes, two parallel cuts were sufficient to successfully produce CNTs. However, with longer tubes and the increasing complexity of arrays and Y-junctions, this was not sufficient anymore. Longer tubes exhibit reduced stability and

are more prone to reattachment to the surrounding bilayer edges. This behavior can be seen in Fig. 1a, where the longer tubes, fabricated by two continuous cuts, reattached to the bilayer. To counteract this effect, the cuts for the subsequent experiments were broken up into smaller 5 nm sections, for example, resulting in the structures shown in Fig. 1b-d. In Fig. 2, a close-up comparison between a nanotube that reattached to the bilayer edge (fabricated by continuous cuts) and a ”free-standing” nanotube (fabricated by sectioned cuts) is given. However, it has to be noted that the comparison between continuous and sectioned cuts is not fully conclusive, as this was not the main aim of the study - besides the cutting sequence, other parameters were varied (tube length and diameter), and the statistics is limited since most of our structures were made with sectioned cuts. For the Y-junctions, the more sophisticated cutting sequence shown in Fig. 3 was employed, where all three tubes are fabricated not only in sectioned cuts but also with cuts alternating between the three tubes, until they join at the center. This minimizes the influence of sample drift on the formation of the junction point. However, Fig. 1d also shows that, while the individual Y-junctions were reproducible using this patterning sequence, some sample drift remained, preventing direct alignment between two neighboring junctions.

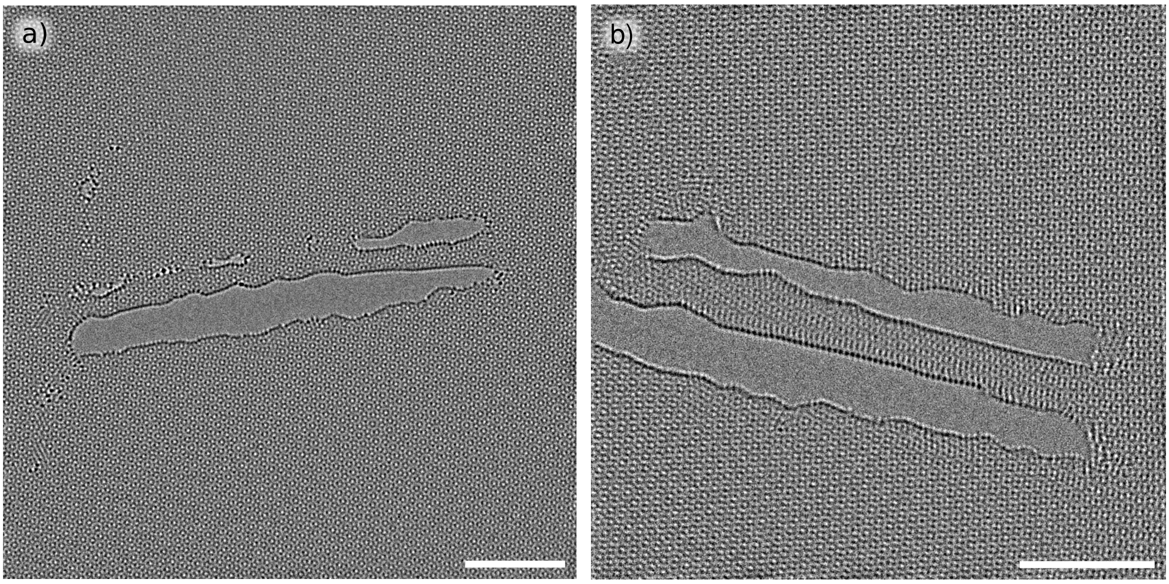

FIGURE 2 a) CNT attached to the bilayer edge, fabricated by two parallel continuous cuts. b) Free-standing CNT, fabricated by parallel 5 nm sections cut alternately. Scale bars are 5 nm.

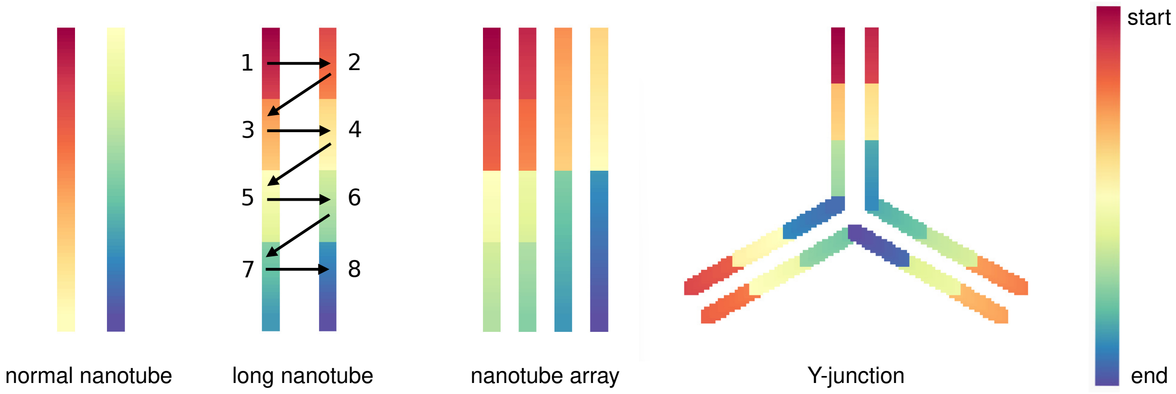

FIGURE 3 Cutting sequences for different structures. Longer cuts were split up into smaller 5 nm sections, carried out alternately to the sides of the nanotube. For the Y-junction, the cuts were arranged in circular sequence.

2. Further Results and Discussion

The relevance of the correct cut angle is demonstrated in Fig.4a. The plot shows the a/b ratio (height/width) of CNTs produced in the molecular dynamics (MD) simulations. Cutting non-twisted bilayer graphene along the zigzag direction resulted in a nearly perfect nanotube with an a/b ratio close to 1. This is because the two ribbons can form a tube without defects in this special case. Subfigure (b) shows a comparison between a 2 and 4 nm nanoribbon, produced in the MD simulations. The side view clearly shows that only the narrow ribbon unfolded into a nanotube. In contrast, the wider ribbon, despite also having merged edges, remained flat. This finding is consistent with the results obtained in the experiment.

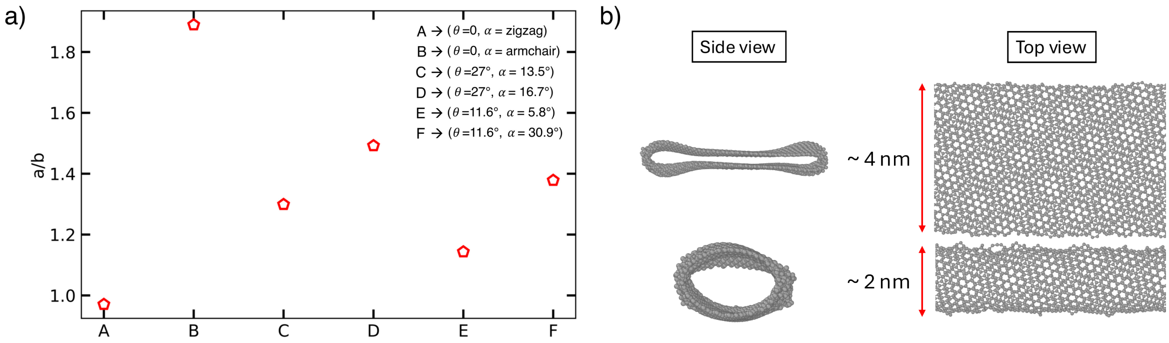

FIGURE 4 a) Plot of nanotubes produced by MD simulations from different cut angles and their respective a/b ratio. The twist angle of the graphene sheets is θ, and α is the cut angle. b) Side and top view of MD simulated nanotubes of different widths. The 4 nm ribbon did not unfold into a nanotube and stayed flat.

Fig.5 shows two HR-TEM images of attempts to fabricate CNTs from widely spaced

cuts. Neither structure unfolded into nanotubes, even though they were written in the same time patterning experiments (see Fig. 1b,c) and with the same line dose as the successful smaller nanotubes. Within (a), the ribbon reattached to the bilayer edge at several points again, and in (b), the gap is completely filled with carbon and heavier contamination atoms. Since the cutting parameters were the same as for the other tubes, we believe that the structure must have had continuous cuts through the graphene bilayer stack at the time of cutting. However, since the nanoribbon was too wide to unfold into a nanotube, it remained flat, and the bilayer edges stayed in proximity to each other. The ribbon edges might then have reattached to the bilayer, closing the gap through a self-healing process mediated by heating.

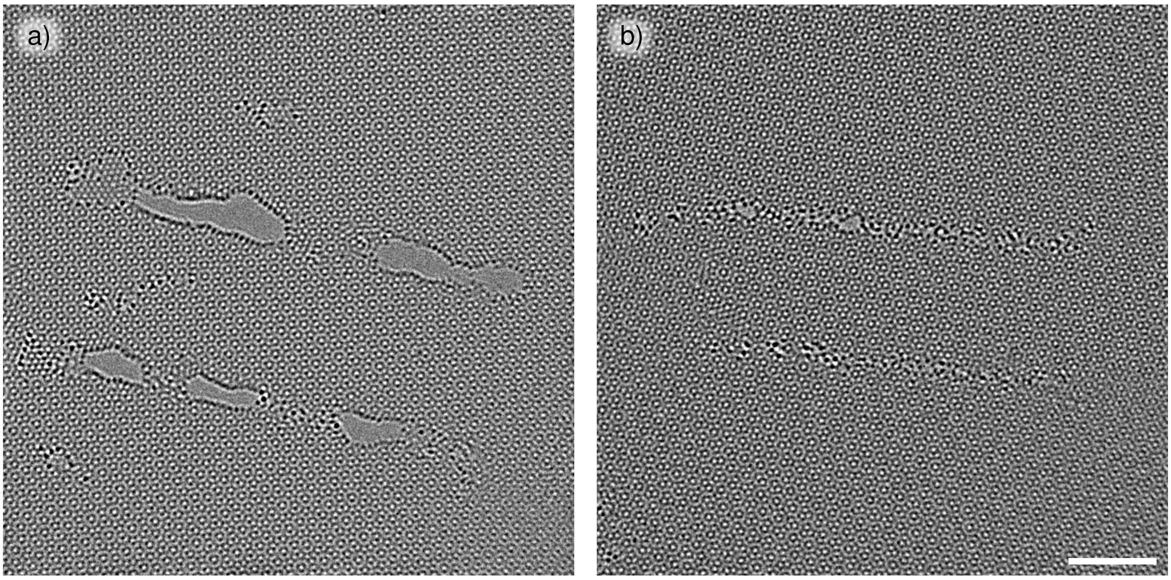

FIGURE 5 HR-TEM images of nanoribbons formed by cuts spaced further apart than 5 nm. Neither of the ribbons unfolded into nanotubes, and even partially merged with the bilayer edge again. Scale bar is 5 nm.

Further examples of healing are shown in Fig.6, where distorted parts of the tube wall and graphene edge become more straight in subsequent images. We attribute this primarily to the elevated sample temperature. However, as the healing occurred over the course of a few seconds during observation, beam-induced effects may also have contributed to the effect.

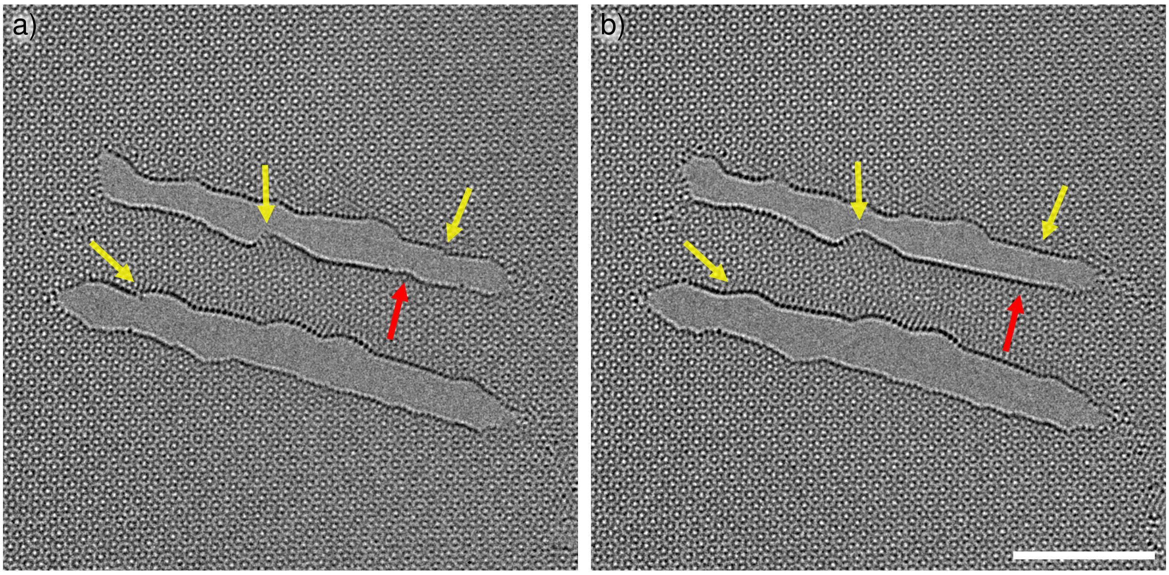

FIGURE 6 Healing of defects within a CNT. a) before and b) after (taken a few seconds apart). The red arrow points at a defective section of the nanotube, which becomes perfectly straight during observation. The yellow arrows point to further defects in the structure, which also appear to undergo a healing process. Scale bar is 5 nm.

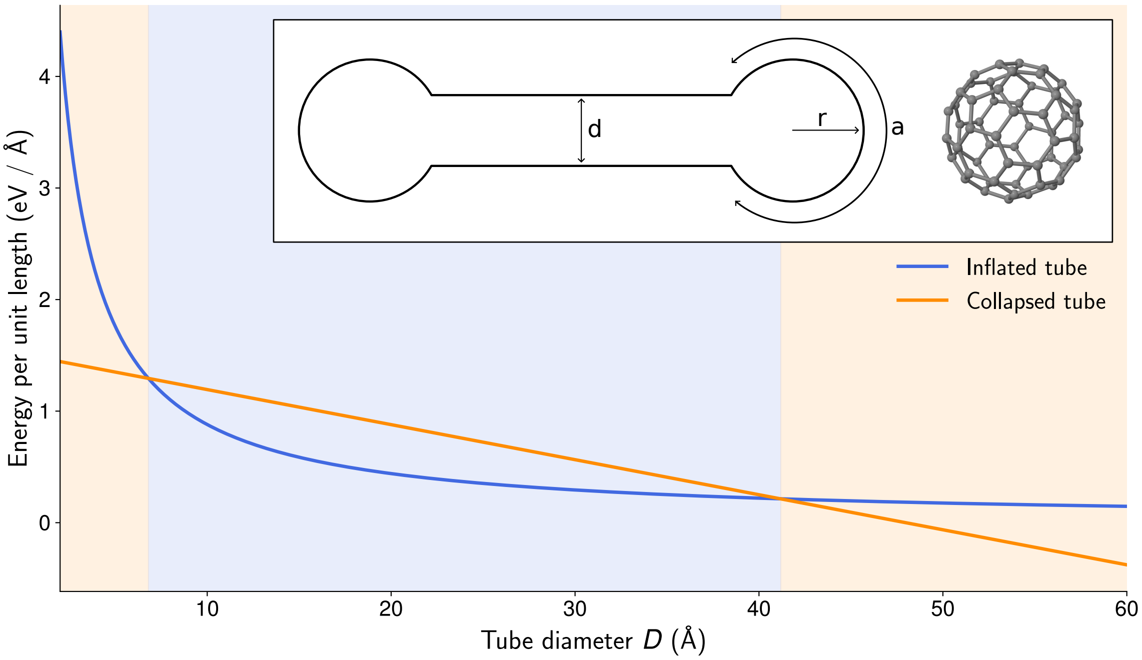

FIGURE 7 Energy per unit length of a CNT as a function of its diameter. The plot compares the energy of an inflated tube, which is modeled by Eq.1, against the energy of the collapsed state, calculated as the sum of Eq.2 and 3. The inset shows an illustration of a collapsed tube with the relevant parameters, with a C_{60} next to it for size comparison.

A quantitative view of the energy consideration between the collapsed and inflated state of a CNT is given in Fig.7. The calculation follows the arguments of Chopra et al. [1] using Eq. 1-3. The increase in energy per unit length of an inflated nanotube

due to the introduction of sp^3 bonding is considered by

$$E_{\text{tube}} = \frac{k\,2\pi R}{2R^2} = \frac{\pi k}{R} \tag{1}$$

with k being the curvature modulus (estimated at 1.44 eV [2]) and R its radius. In contrast, Eq. 2 and 3 describe the collapsed ribbon, where Eq. 2. describes the bending energy at the bilayer edge, and Eq. 3 the attractive van der Waals (vdW) interaction:

$$E_c = \frac{2ka}{2r^2} = \frac{ka}{r^2} \tag{2}$$

$$E_v = -E_{\text{vdW}}\,(\pi R - a) \tag{3}$$

For the calculation shown in Fig. 7, the radius of curvature of the closed bilayer edge r was set to be 3.5 Å(comparable to values acquired by [3]), which equals the radius of atom positions of a C_{60} fullerene. The arc length of the curved edge region a is approximated with $a = r\pi$. E_{vdW} was assumed to be ~0.02 eV/Å^2 [4]. The model assumes that $a, r << R$. Furthermore, a and r are independent of R. Under this assumption, it has to be noted that the apparent lower threshold diameter below ~ 0.7 nm lies outside the validity range of the model. In contrast, an upper critical tube diameter of 4.1 nm is obtained, above which nanotubes are energetically favored to collapse into flat ribbons. This is in good agreement with our MD simulations and experimental observations.

References

[1] Nasreen G Chopra et al. “Fully collapsed carbon nanotubes”. In: Nature 377.6545 (1995), pp. 135–138.

[2] Yujie Wei et al. “Bending rigidity and Gaussian bending stiffness of single-layered graphene”. In: Nano Lett. 13.1 (2013), pp. 26–30.

[3] Maoshuai He et al. “Precise determination of the threshold diameter for a single-walled carbon nanotube to collapse”. In: ACS Nano 8.9 (2014), pp. 9657–9663.

[4] L A Girifalco and R A Lad. “Energy of cohesion, compressibility, and the potential energy functions of the graphite system”. In: J. Chem. Phys. 25.4 (1956), pp. 693–697.
